# Reflecting on the Germanwings Disaster: A Systematic Review of Depression and Suicide in Commercial Airline Pilots

**DOI:** 10.3389/fpsyt.2018.00086

**Published:** 2018-03-20

**Authors:** Terouz Pasha, Paul R. A. Stokes

**Affiliations:** ^1^Faculty of Life Sciences and Medicine, King’s College London, London, United Kingdom; ^2^Centre for Affective Disorders, Institute of Psychiatry, Psychology and Neuroscience, King’s College London, London, United Kingdom

**Keywords:** commercial aviation, mental health, mood disorders, pilot, suicide

## Abstract

**Background:**

The 2015 Germanwings Flight 9525 disaster, in which 150 people were killed after the co-pilot may have intentionally crashed the plane in a suicide attempt, highlights the importance of better understanding the mental health of commercial airline pilots. However, there have been few systematic reviews investigating the topic of mental health in commercial aviation. This systematic review aims to identify the types and prevalence of mental health disorders that commercial airline pilots experience with a focus on mood disorders and suicide risk.

**Methods:**

A systematic literature search was performed using PubMed, EMBASE, and PsycINFO databases. Eligible studies were assessed and data was extracted and analyzed.

**Results:**

20 studies were identified. The prevalence of depression experienced by commercial airline pilots in this review ranged from 1.9% to 12.6%. Factors that negatively impacted the mental health of pilots included substance abuse, experiencing verbal or sexual abuse, disruption in sleep circadian rhythms and fatigue.

**Discussion:**

This systematic review identifies that commercial airline pilots may experience depression at least as frequently as the general population. Commercial airline pilots experience occupational stressors, such as disrupted circadian rhythms and fatigue which may increase risks of developing mood disorders. Most studies identified in this review were cross-sectional in nature with substantial limitations. There is a clear need for further higher quality longitudinal studies to better understand the mental health of commercial airline pilots.

## Introduction

Commercial airline pilots undergo rigorous selection and training and, once qualified, require frequent competency checks, including annual medical reviews for the entire duration of their career. Aviation safety is rarely threatened by the deliberate destruction of airplanes by pilots, and when they do occur, events are often shrouded with uncertainty as to the pilot’s motivation. The 2015 Germanwings Flight 9525, in which the co-pilot may have locked the captain out of the cockpit and crashed the plane, killing 150 passengers and crew, has highlighted the importance of better understanding the mental health of commercial airline pilots. A final investigative report revealed evidence that the co-pilot experienced a psychotic depressive episode that started in 2014 and lasted until the day of the accident (1). The Germanwings disaster is not an isolated incident. Notable cases of possible murder-suicide by commercial airline pilots, such as the 1999 EgyptAir crash which killed 217 people (2) and the 1997 SilkAir Flight 185 crash which killed 104 people (3), highlights that optimizing the medical and psychological health of pilots is important for avoiding accidents and fatalities.

While aircraft-assisted suicide is rare, suicide and mood disorders are not. Over 90% of suicide victims experience at least one major mental disorder, with major depressive disorder (MDD) being the most common (56–87%) (4). MDD is one of the leading causes of chronic disability (5) and affects 350 million people worldwide (6).

The health of commercial airline pilots is assessed annually, and pilots licenses and flying privileges may be suspended if health problems are detected and reported to civil aviation authorities. In the UK, the Civil Aviation Authority regulates and implements medical requirements for pilots, including mental health. The U.S. Federal Aviation Administration (FAA) and European Aviation Safety Regulation stipulate similar criteria on mental health. Although pilots with a history of mood disorders can be licensed, pilots should have “no established medical history or clinical diagnosis of any psychiatric disease or disability, condition or disorder, acute or chronic, congenital or acquired which is likely to interfere with the safe exercise of the privileges of the applicable license(s)” (7). Additionally, those dependent on any psychotropic substances are considered unfit until full recovery is achieved (7).

Overall, there have been few studies identifying the types of mental health problems experienced by pilots. This systematic review will identify and summarize studies investigating the prevalence and underlying causes of mental health disorders experienced by commercial airline pilots, with a focus on mood disorders and suicide.

## Methods

### Search Strategy

The search was designed to identify the mental health factors in commercial aviation pilots, including risk factors, prevalence, types of mental health disorders, and aircraft-assisted suicide. The Preferred Reporting Items for Systematic Reviews and Meta-Analyses (PRISMA) statement was used to provide the structure of this review. A comprehensive search was performed using a combination of MEDLINE, EMBASE, and PsycINFO databases. Citation lists of relevant studies were searched to check for any additional papers. The search is up-to-date through February 2017.

The following Medical Subject Headings or text word terms was used:

The following search string was used: [(airline pilot$) OR (commercial pilot$) OR (aircraft pilot$) OR (aviation) OR (aerospace)] AND [(mental health) OR (depres*) OR (mood disorder) OR (mental disorder$) OR (depressive disorder) OR (panic) OR (suicide)].

### Eligibility Criteria

The inclusion criteria comprised any original peer-reviewed studies in the English language characterizing the mental health of commercial aviation pilots. Participants included any commercial aviation pilots worldwide who held a qualified license under their respective regulatory board. Interventions and comparisons were not relevant. Studies required at least one outcome measure which was categorized in: factors contributing to poor mental health in pilots, prevalence of mental health disorders, suicide and substance abuse.

The exclusion criteria were single-case studies, reviews, meta-analysis, books, research letters, commentaries, studies with non-civil aviation pilots as participants, studies assessing aircraft safety, and studies focused on non-psychiatric medical conditions of pilots, such as cardiovascular conditions.

## Results

**Figure 1:**
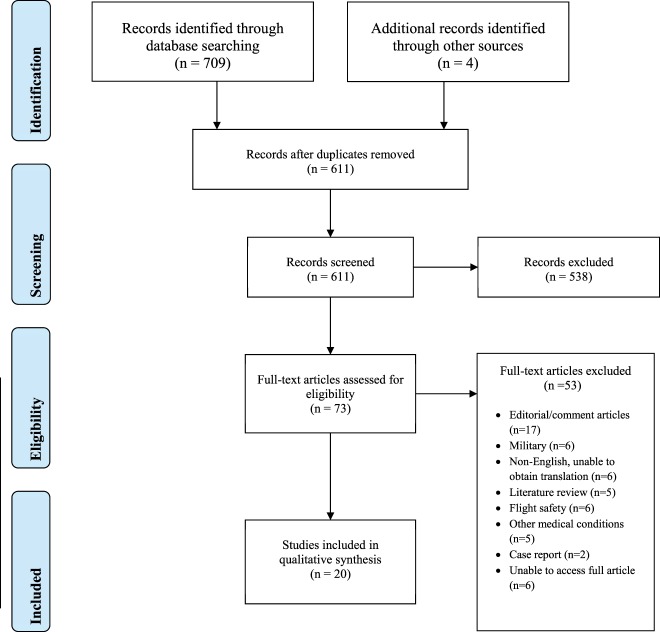
Preferred reporting items for systematic reviews and meta-analyses flow diagram of included studies.

Figure 1 represents the studies identified at each phase of our search using the PRISMA flow diagram. The search strategy identified 709 papers of which 20 met the inclusion criteria (8–27). We divided the studies into three groups, displayed accordingly: depression and associated psychosocial factors (Table 1), suicide and substance abuse (Table 2), and sleep and fatigue (Table 3).

**Table 1:** Summary of included studies on depression and associated psychosocial factors experienced by commercial aviation pilots (reverse chronological order).

Reference	Aim	Period of data collection	Sample size	Country population	Design	Outcomes of interest
**Mental health disorders and psychosocial stressors**						
Wu et al. (8)	To describe airline pilot mental health—with a focus on depression and suicidal thoughts	April–December 2015	1,837	Multinational^a^	A descriptive cross-sectional study *via* an anonymous web-based survey	13.5% met the depression PHQ-9 score of ≥10. 4.1% had suicidal thoughts within the past 2 weeks. Higher depression levels found in those using sleep-aid medication and who experience sexual or verbal harassment
Feijó et al. (9)	To estimate the prevalence of suspected cases of CMD on Brazilian civil aviation pilots and their associations	October 2009 and October 2010	755	Brazil	A quantitative cross-sectional study using a self-administered anonymous questionnaire	CMD prevalence was 6.7%. Variables relating to workload and the practice of physical activity were significantly correlated with the estimate of CMD
Sykes et al. (10)	To investigate the health of the pilot population of an Oceanic-based airline compared to the health of the general population	November 1, 2009–October 31, 2010	595	New Zealand	Medical questionnaire was completed by pilots at their medical certificate renewal	Most medical conditions, including depression, pilots had a lower prevalence when compared to the general population
Widyahening (12)	To identify the effect of work stressors and other factors on mental-emotional disturbances among airline pilots	May–July 1999	109	Indonesia	A cross-sectional study of questionnaires provided to pilots during their routine medical examination	The prevalence of mental-emotional disturbances was 39.4%. Those with high levels of work stressors had a risk of 4.6 times higher mental-emotional disturbances vs control group
Little et al. (11)	To investigate whether an airline corporate instability is related to pilots’ stress symptoms	Not discussed	432	United States	Symptoms of stress questionnaire was administered to three random samples of pilots	Pilots employed by the airline with a history of corporate instability reported significantly higher stress and depression symptoms than control group.
Cooper and Sloan (26)	To investigate the sources of occupational and domestic stress, together with life events and coping strategies	Not discussed	442	England	Postal Survey	Mental ill-health was found to be associated with lack of autonomy at work, fatigue, and flying patterns together with an inability to relax and lack of social support
**Use of antidepressants**						
Sen et al. (16)	Follow-up study examining whether pilots with detected SSRI in fatalities had disqualifying psychological conditions and/or reported use of antidepressants	1990–2001	61, 59 of which had medical records in database	United States	FAA’s and NTSB’s Aviation Accident Database	Disqualifying psychological conditions self-reported in 7/59 (12%). Such conditions and/or drug use was not reported in the remaining 52 (88%) of pilots.From personal medical records, 12/61 pilots (20%) had a psychological condition and/or used selective serotonin receptor inhibitors (SSRIs)
Akin and Chaturvedi (15)	Examine the presence of SSRIs in pilot fatalities	1990–2001	4,184	United States	CAMI Toxicology Database	61/4,184 (1.5%) civil aviation accidents had pilots with SSRIs. 33% of these, other drugs and ethanol was detected. SSRIs were a contributory factor in at least 9/61 accidents

*^a^Participants from >50 different countries*.

*PHQ-9, Patient Health Questionnaire-9; CMD, common mental disorder; FFA, Federal Aviation Administration; NTSB, National Transportation Safety Board; CAMI, Civil Aerospace Medical Institute; SSRI, selective serotonin reuptake inhibitor*.

**Table 2:** Summary of included studies on suicide and substance abuse in commercial aviation pilots (reverse chronological order).

Reference	Description	Method	Time frame of accidents	Sample size	Sample specification	Presence of drugs (excluding ethanol) in system	Presence of ethanol in system	Stressors identified
Politano and Walton (13)	Analysis of NTSB Aircraft-Assisted Pilot Suicides	NTSB’s accident database (eADMS)	1983–2014	51	100% Male, mean age 38 years	15.7% were on prescription medication and 3.9% on non-prescription medication	13.70%	27. 5% relationship issues, 7.6% legal issues (total of 54.9% had some sort of stressor)
Chaturvedi et al. (17)	Sampled pilots in civil aviation accidents during 1983–2013 for ethanol and drugs and compared the data in 5-year cohorts (1989–1993, 1994–1998, 1999–2003, and 2004–2008)	CAMI and NTSB’s database	1989–2003	1,169	Not discussed	Only drugs were found in 45% (523/1,169) of cases. 48% of airmen had drugs with or without ethanol in their system	7.3% (85) had ethanol in their system. 3.3% (38) had both ethanol and drugs in their system	Not discussed
Vuorio et al. (27)	Follow-up study of Lewis et al. to provide a new estimate for the overall aircraft-assisted suicide over a 20-year period	NTSB database^a^	2003–2012	8	100% Male, mean age 4 years	7/8 cases had a toxicological analysis. Of these, 3/7 used antidepressants	4/7 had ethanol in their system	Personal, legal, and/or relationship problems were mentioned in 4/8 investigations. Suicidal ideation and/or previous suicide attempts made in 5/8 cases
Lewis et al. (18)	10-year review of epidemiological and toxicological findings from aircraft-assisted pilot suicides	NTSB, CAMI’s database, and DIWS	1993–2002	15^b^	100% Male, median age 40 years	2 tested positive for benzodiazepines, 1 for marijuana, 1 for cocaine, and 1 for venlafaxine	4 (26.7%)	40% had domestic difficulties
Bills et al. (14)	Comparative analysis on characteristics of aviation-assisted suicides vs non-suicidal aviation accidents	NTSB database	1983–2003	37	100% Male, 48.6% <39 years old, 51.4% >40 years old	21.6% on prescription drugs, 13.5% on illegal drugs	24.30%	45.9% domestic and social problems, 40.5% legal issues

*NTSB, National Transportation Safety Board; CAMI, Civil Aerospace Medical Institute; DIWS, Aerospace Medical Certification System*.

*^a^This study contained a literature review and data collection from the NTSB database. Only figures from the NTSB database has been extracted from this study*.

*^b^16 if including the pilot who threw himself out which did not result in an accident*.

**Table 3:** Summary of included studies investigating sleep and fatigue in commercial aviation pilots (reverse chronological order).

Reference	Aim	Sample size	Design	Outcomes of interest
Sallinen et al. (19)	What airline pilots do to maintain their alertness while being on duty and association of sleep and alertness	90	Sleep was measured by a diary and actigraphs, on-duty alertness by the Karolinska Sleepiness Scale in all flight phases, and on-duty alertness management strategies by the diary	Short- and long-haul flight duty Perios covering the whole domicile night (00:00–06:00 at home base) were most consistently associated with reduced sleep-wake ratio and subjective alertness
O’Hagan et al. (22)	Investigating work hours and their associated factors’ contribution to mental health issues among pilots	701	Anonymous online survey	Pilots who reported typically spending longer hours on duty per week were twice as likely to report feeling depressed or anxious
Reis et al. (23)	To provide the first prevalence values for clinically significant fatigue in Portuguese airline pilots	456	Questionnaires placed in pilots’ personal lockers including a self-response fatigue severity sale	The prevalence values for total and mental fatigue achieved in the Portuguese airline pilots were: 89.3% (long) and 94.1% (medium/short)
McLaughlin et al. (25)	To assess seasonal effects on shift-work tolerance	88	Questionnaires were completed twice, near the summer and winter solstices	General psychological health and mood were significantly worse in winter, while sleep was more disturbed in summer. In winter, 31% exceeded the cut-off for psychological distress and >70% scored higher than normal range for depressive symptoms
Jackson et al. (20)	How much subjective fatigue short-haul pilots report, comparing low-cost and scheduled airline pilots	162	Anonymous questionnaires posted on the Professional Pilots’ Rumors Network website	75% reported severe fatigue. Reported more frequently by low-cost airline pilots and of higher rating than scheduled airline pilots
Petrilli et al. (21)	To investigate pilots’ amount of sleep, subjective fatigue, and sustained attention before and after international flights	19	Pilots given wrist activity monitors and completed sleep and duty diaries	Sleep in the previous 24 h was a significant predictor of self-rated fatigue and mean response speed after the international flight sectors
Bourgeois-Bougrine et al. (24)	To clarify what fatigue means to pilots on short- and long-haul flights	739	Questionnaires were distributed to pilots through four airlines	Self-reported manifestations of fatigue in 60% of LHF pilots and 49% of SHF pilots

### Depression

#### Prevalence and Demographics

**Figure 2:**
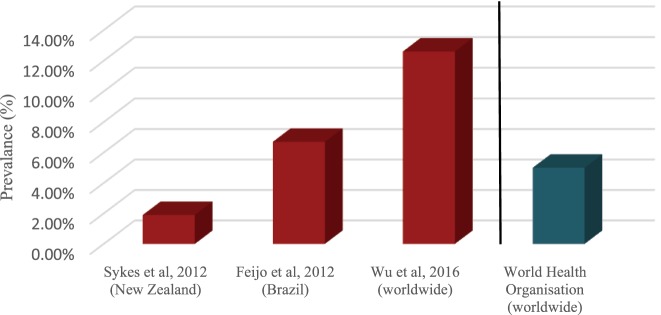
Prevalence of depressive disorders in aviation pilots vs depression in general population.

Sykes et al. who evaluated New Zealand airline pilots in a non-anonymous national survey, found the prevalence of depression to be 1.9% (10). Feijó et al.’s anonymous national study, found prevalence of pilots with common mental disorders (CMD), such as mixed anxiety and depression, to be 6.7% (9). Conversely, Wu et al. anonymously assessed pilots internationally through the Patient Health Questionaire-9 (PHQ-9) threshold for MDD, and yielded a much higher prevalence of 12.6% (8). The National Institute of Mental Health estimates that 6.9% of the U.S. population experience depression (28) and the World Health Organization states a 5% prevalence of depression worldwide (29). Comparing this to Wu et al.’s results, the most reliable prevalence study of the three, indicates that a higher prevalence rate is experienced by commercial airline pilots when compared to the general population (Figure 2).

Wu et al. found commercial airline pilots median depression score to decrease with increasing age (8). However, Feijó et al. reported that pilots >35 years old have a higher prevalence of CMD (7.2%) than those ≤35 years old (4.5%) (9). This may be due to older pilots being less inclined to admit to experiencing CMDs. This is demonstrated by Sykes et al. who found that with increasing age, the percentage of pilots who attend the airline’s medical unit for their medical certificate renewal decreases (10).

With regards to gender, results from both anonymous surveys in Table 1 reveal female pilots to be more likely to be diagnosed with depression in their lifetime (4.7% females vs 2.9% males) (8) and have a higher prevalence of CMD (7.7% females vs 6.6% males) (9). However, Feijó et al.’s study involved a significantly lower number of total female pilots compared to male participants (13 vs 742) which may have affected the results (9).

#### Factors Associated With Depression

Several other factors are associated with mental health problems in commercial airline pilots. Wu et al.’s found that 16.2% of pilots consuming more than one drink of alcohol a day, met the PHQ-9 threshold for MDD (8). A high proportion of pilots who experienced sexual and verbal harassment at least four times a week also met the threshold for MDD (36.4 and 42.9%, respectively). Additionally, the proportion of pilots meeting the depression threshold, within the past month, was higher with increased frequency of sleep-aid medicines use (8). Feijó et al. found that workload and regular physical activity was significantly associated with CMD. Pilots who exercised regularly and had a light workload experienced a CMD prevalence rate of only 2.2% compared to 31% of pilots with heavy workload and no physical activity (9).

Cooper and Sloan investigated coping strategies of commercial airline pilots, and found lack of autonomy at work, fatigue, and lack of social support to be strongly associated with mental ill-health (26). An Indonesian study showed that length of employment and total flight of ≥10,000 h is a strong risk factor for emotional stress among Indonesian pilots (12). Additionally, a 1990 study found that pilots reported “significantly more stress and depression symptoms” if employed by an airline with a history of corporate instability compared to pilots from stable airlines (11).

#### Use of Antidepressants

The remaining studies in Table 1 focus on selective serotonin receptor inhibitors (SSRIs) toxicological findings in aviation accidents in United States (U.S.). Aeromedical regulatory authorities have previously been reluctant to allow pilots to take SSRIs due to its potential effect on the central nervous system (15). At the time of the 2003 study, SSRI’s were not approved for use by U.S. commercial airline pilots, yet the study discovered the presence of SSRIs in 61 pilots involved in aviation accidents during 1990–2001 (15). Out of these pilots, 39 had additional drug(s) and/or alcohol within their system. Additionally, the majority of these pilots had not disclosed their psychiatric condition (88%, 56/59) and 95% (56/59) of the same pilots had never reported the use of an antidepressant (15, 16).

### Suicide

Table 2 presents studies investigating suicide and toxicological findings in aviation accidents. Pilots in the U.S. who are fatally injured in civil aviation accidents are toxicologically evaluated at the FAA’s Civil Aerospace Medical Institute (CAMI) upon an authorization granted by the NTSB. All U.S. studies included in Table 2 used the NTSB’s database for its data collection and analysis. Politano and Walton’s data, however, analyzed the largest range from 1983 to 2014 making their results the most accurate and up-to-date (13).

#### Prevalence of Suicide

Suicidal ideation is a key risk factor for future completed suicide. According to the NTSB database, 7.8% of pilots who committed suicide had made a previous suicide attempt (13). The most contemporary statistic of commercial airline pilot suicidal ideation comes from Wu et al., where 4.1% of pilots had thoughts of “being better off dead” or self-harm within the past 2 weeks of the survey (8). However, suicidal ideation does not necessarily lead to a suicide attempt. Regarding fatal aircraft accidents, between 1993 and 2012 U.S. aircraft-assisted suicide by pilots totals 0.33% (24/7,244 fatal accidents) (18, 27).

#### Factors Associated With Suicide

Politano and Walton found the average age of U.S. pilots committing aircraft-assisted suicide to be 38 years (13). One case-controlled study found that compared to non-suicidal accident controls, suicide incidents involved younger pilots with pilots younger than 40 being five times more likely to crash due to suicide than pilots aged 40 or older (13). All studies investigating pilot-assisted suicide found all pilots to be male (Table 2).

The NTSB database contains data about any pilot involved in accidents and subsequent investigative reports on stressors, the pilot may have experienced around the time of the accident. Politano and Walton found that 54.9% of the 51 pilots had an identifiable stressor established by NTSB. Of these, 27.5% were relationship issues, 7.6% were legal problems, and 5.9% dealt with a serious illness of the pilot or a close relationship (13). Another study found that, of the 37 pilots, 51% left a suicide note and that most suicide cases occurred between October and March (62%) (14).

### Substance Use Disorders

Under the FAA regulation, ≥40 mg dl (21) is the blood alcohol concentration in which no person may operate or attempt to operate an aircraft. Since all the toxicological studies in Table 2 used the same NTSB/CAMI database, it would be reasonable to assume that they employed FAA’s ≥40 mg dl (21) cut-off point. Chaturvedi et al.’s most recent 5-year cohort of pilots involved in civil aviation accidents (2004–2008) revealed that 7% of pilot fatalities had detectible alcohol within their system. This is a similar result to the previous 5-year cohorts (1989–2003) suggesting that alcohol usage has not changed through the years (17). Further, Politano and Walton found that 13.7% of pilots involved in fatal incidents had alcohol in their system post-mortem (13) which is lower than the 32.39% of alcohol found in the general population (30). However, the combined presence of alcohol and drugs in fatally injured pilots went up by 239% (17). This overall increase, the authors stated, was largely attributed to the continuous rise in the use of prescription drugs and the increase in capabilities of laboratories to detect drugs. This is consistent with their results which saw prescription drug use rise to 583% between 1989 and 1993 (6%) and 2009 and 2013 (35%) (17). Additionally, out of all aircraft-assisted suicides in the U.S. between 1983 and 2014, 15.7% were on prescription medication vs 3.9% on non-prescribed medication (13).

### Sleep and Fatigue

Studies exploring the relationship of circadian sleep cycles and fatigue in commercial aviation pilots within both long- and short-haul flight are collated in Table 3.

A recent anonymous online survey revealed that pilots who reported working longer hours per week were twice as likely to report feeling depressed or anxious (22). However, this finding was only significant for pilots working 36–40 h after which the likelihood of reporting depression or anxiety progressively decreased. The authors speculated that the pilots’ experiences of job-related sleep disturbance and fatigue may be the reason for this (22).

Several studies in Table 3 revealed high proportions of pilots reporting fatigue (20, 23, 24), with pilots flight duty periods covering whole domicile nights being at particular risk (19). One Portuguese study found mental fatigue to be as high as 89.3% in pilots who work on long-haul flights and 94.1% in medium/short-haul flights (23). While another study found 60% of pilots flying long-haul routes and 49% of pilots flying short-haul routes reported fatigue (24). Other than the length of flight, Craig et al. observed low-cost airline pilots to report fatigue more frequently and of a higher rating than scheduled airline pilots (20).

Seasonal variation was shown to influence pilot’s mental health and sleep patterns. In the winter, 31% of pilots were found to exceeded the cut-off for psychological distress, and >70% scored in the higher than normal range for depressive symptoms. While pilots during the winter reported lower mood and worse mental health, participants reported worse sleep in summer (25).

## Discussion

This systematic review has identified that commercial airline pilots are at similar or potentially increased risks of experiencing depression as the general population. Additionally, pilots experience several occupational stressors such as disrupted circadian rhythms and fatigue, which are recognized as being associated with the development of mood disorders (31). Other factors identified in this review as potentially being associated with depression in commercial airline pilots are high workload, experiencing verbal and sexual harassment, and corporate instability of airline companies.

Prevalence of depression within commercial aviation in this review ranged from 1.9 to 12.6% (8–10). This is much higher than the reported prevalence of MDD in military pilots (0.06%) (32). The large disparity in results between anonymous and non-anonymous studies may be due to fear of pilots negatively impacting their own careers. Arguably the most accurate study is of Wu et al. as they anonymously assessed pilots from over 50 countries. Coincidentally, they also observed the highest prevalence of both MDD (12.6%) and suicidal ideation (4.1%) in commercial pilots. This may be an underestimation as lower participation among pilots with severe depression is expected. These figures oppose previous beliefs that the mental health of commercial airline pilots is better than the general population. This also implies that potentially hundreds of active pilots currently flying may be experiencing unreported mental health disorders, including suicidal ideation.

Due to the overall low incidence of aircraft-assisted suicide by pilots, identifying factors that may predispose a pilot to crash a plane is challenging, especially as studies investigating this were retrospective and cross-sectional. Air deaths from pilot suicide may also be underreported. From the NTSB database, we know that investigators do not always determine what causes crashes as it is not always clear whether an accident was the result of a deliberate act, dysfunction of machinery, or human error. However, from the data we do have, 100% of aircraft-assisted suicide pilots are male which would be consistent with previous research that men use more violent avenues to end their lives (33–35). This may also be due to there being a small number of female pilots in general, limiting female accessibility to studies.

Toxicological post-mortem studies identified in this review show that prescription drug use in fatally injured pilots rose from 583% from 1989 to 2013, while alcohol use remained similar throughout the same dates (17). However, sources of blood specimens are frequently unknown due to the severe impact of trauma within aviation accidents. Therefore, blood concentrations of any substances found post-mortem in these pilots may not necessarily represent their antemortem levels and so interpretation of these results should be handled with caution. Additionally, out of the pilots involved in aviation accidents which had SSRI detected within their bodies, 88% had not disclosed their psychiatric condition and 95% had never reported the use of antidepressants in the first place (15). These results support concerns of pilots underreporting adverse mental health symptoms for fear of negative career impact.

This review found that pilots experience high levels of self-reported fatigue (23) and this is shown, together with job-related sleep disturbance, to be the main cause of higher rates of feeling depressed or anxious in longer hours on duty (22). While research is still exploring the relationship between mental health and sleep, previous studies have shown that sleep deprivation has a significant effect on mood (36). A pilot’s lifestyle of shift work, night work, and time zone changes may not be conducive with regular sleeping patterns. To date, studies have focused more on the safety impact of fatigued pilots, and have focused less on the mental health impacts of fatigue experienced by commercial airline pilots. More research is required in investigating the long-term effect of sleep deprivation and fatigue on the mental health of commercial pilots.

There were substantial limitations within the studies included in this review. Due to the wide heterogeneity between the objectives and study methods of identified papers, a qualitative analysis was not feasible. Most studies relied on retrospective self-reporting data that was cross-sectional in nature. This may cause biased results due to participants potentially by misunderstanding questions as well as relying on their recall. Because data collection was carried out at a single point in time, it is unclear whether pilot exposure to a psychosocial stressor occurred before, after, or during the onset of their mental health outcome. It is, therefore, difficult to infer causality. Studies using the NTSB databases for data looked at all civil aviation pilots which includes both private pilots and commercial airline pilots. Often, the distinction between the two groups was not made in the results. Survey-based studies used different rating methods, focused on different mental health disorders, and some were anonymous while others not. Studies that conducted anonymous surveys did not conduct medical interview follow-ups to confirm a diagnosis of a mental health disorder, nor did they have access to medical records. However, this would be challenging to do, as it may sacrifice the anonymity of the pilot. Overall, the number of studies included in this systematic review was small and most of the studies themselves had small sample sizes, limiting the quality of conclusions that can be drawn.

Although mental health within commercial aviation is not a new topic, the ability to identify and help pilots who struggle with mental health problems remains a present-day challenge. From this review, the paucity in high quality research in mental health within commercial aviation is clear. Future studies should emphasize anonymous population-based longitudinal research with the aim of identifying contributing factors to poorer mental health outcomes within aviation pilots. Refinement of civil aviation databases should be considered to improve quality of data collection. This will lead to improvement in understanding the mental health problems experienced by commercial airline pilots which is a key for the development of regulations, improving mental health support for pilots, and intervening before mental health symptoms interfere with aircraft operations.

## Author Contributions

TP drafted the manuscript. PS reviewed draft manuscripts and revised the final version of the manuscript.

## Conflict of Interest Statement

TP has no relevant conflicts to declare. PS has received support for research, expenses to attend conferences, and fees for lecturing and consultancy work (including attending an advisory board) from life sciences companies, including Corcept Therapeutics, Indivior, and Liva Nova. PS is a consultant psychiatrist within a tertiary level specialist and an enhanced secondary level affective disorders service, and a specialist consultant advisor in mood disorders to the UK Civil Aviation Authority. The reviewer TM and handling editor declared their shared affiliation.
